# Health-related quality of life instruments in pediatric disorders of gut–brain interaction: a COSMIN-based systematic review

**DOI:** 10.1186/s41687-026-01093-2

**Published:** 2026-05-22

**Authors:** Zehui Zhao, Yueyue Chen, Huiyu Zeng, Yuqi Fang, Siyuan Hu

**Affiliations:** 1 First Teaching Hospital of Tianjin University of Traditional Chinese Medicine, Tianjin, China; 2 National Clinical Research Center for Chinese Medicine, Tianjin, China

**Keywords:** Disorders of gut–brain interaction, Functional gastrointestinal disorders, Health-related quality of life, Pediatric, Patient-reported outcome measures, COSMIN, Systematic review

## Abstract

**Background:**

Functional gastrointestinal disorders, now referred to as disorders of gut–brain interaction (DGBI), impose substantial functional and psychosocial burdens on affected children and their families. Health-related quality of life (HRQoL) has become an important patient-reported outcome in this field. This review aimed to systematically evaluate the measurement properties of instruments used to assess HRQoL in pediatric DGBI.

**Methods:**

A systematic review was conducted following the COSMIN methodology. PubMed, Embase, and Web of Science were searched from database inception to Oct 23, 2025. Studies evaluating the measurement properties of patient-reported outcome measures assessing HRQoL in children aged 0–18 years with disorders of gut–brain interaction were included.

**Results:**

14 studies evaluating nine health-related quality of life instruments were included. These comprised four generic instruments (PedsQL 4.0, EQ-5D-Y, CHU-9D, and the PedsQL Family Impact Module) and five disease-specific instruments (PedsQL Gastrointestinal Symptoms Scales, PedsQL Gastrointestinal Worry Scales, Infant Colic Questionnaire, PedFCQuest-PR, and the Defecation Disorder List). The instruments were evaluated across six pediatric DGBI subtypes: cyclic vomiting syndrome, infant colic, functional dyspepsia, irritable bowel syndrome, functional abdominal pain—not otherwise specified, and functional defecation disorders.

**Conclusion:**

Among generic instruments, PedsQL 4.0 has the most comprehensive supporting evidence and can be used when a broad assessment of health status is required. Disease-specific instruments, particularly the PedsQL Gastrointestinal Symptoms Scales and the PedsQL Gastrointestinal Worry Scales, align more closely with gastrointestinal-related impacts and may be better suited for evaluating DGBI-related outcomes. Evidence for several measurement properties remains incomplete, especially for structural validity and responsiveness. More systematic evaluation of these instruments will help improve outcome measurement and strengthen the interpretation of treatment effects in pediatric DGBI.

**Supplementary information:**

The online version contains supplementary material available at 10.1186/s41687-026-01093-2.

## Introduction

Functional gastrointestinal disorders (FGIDs), now termed disorders of gut–brain interaction (DGBI), are among the most prevalent chronic gastrointestinal conditions in childhood. This terminology was introduced in Rome IV and further reinforced in Rome V, reflecting a shift from defining these disorders by the absence of structural abnormalities to understanding them as symptom-based disorders involving altered gut–brain regulation [1]. Community-based studies suggest that approximately one quarter of children and adolescents aged 0–18 years meet criteria for at least one Rome IV diagnosis [2]. Although DGBI lack identifiable structural or biochemical abnormalities, their clinical impact is far from benign. Recurrent abdominal pain, nausea, vomiting, and altered bowel habits often lead to persistent symptom burden, psychological distress, activity limitations, school absenteeism, increased healthcare utilization, and substantial caregiver strain [3, 4]. Their impact frequently extends beyond the affected child, disrupting family functioning, parental productivity, and overall household well-being.

This multidimensional burden underscores the importance of health-related quality of life (HRQoL) as a critical outcome domain. By integrating symptom severity with functional impairment and psychosocial well-being, HRQoL reflects the broader effects of disease on daily functioning, emotional health, and social participation—dimensions not fully captured by symptom-based indices alone. As patient-centered outcomes assume increasing prominence in clinical research, regulatory evaluation, and health technology assessment, the selection of appropriate HRQoL instruments carries direct implications for trial design, interpretation of therapeutic benefit, and evidence synthesis [5].

Despite this growing recognition, clear operational guidance on the selection of HRQoL instruments for pediatric DGBI remains lacking. Determining whether an instrument is suitable for this population requires rigorous evaluation of its measurement properties within the intended context of use. Reliance on historical precedent or frequency of prior application is insufficient; instrument choice must instead be supported by transparent and methodologically sound evidence. In the absence of such evaluation, outcome assessment risks conceptual misalignment, measurement bias, and misinterpretation of longitudinal change.

The COSMIN (COnsensus-based Standards for the selection of health Measurement INstruments) methodology provides a comprehensive and standardized framework for the appraisal of patient-reported outcome measures. Widely endorsed for systematic reviews of patient-reported outcome measures (PROMs), COSMIN offers a structured taxonomy of measurement properties, validated risk-of-bias checklists, explicit criteria for adequacy, and a modified GRADE approach for evidence synthesis. This framework facilitates transparent, reproducible, and population-specific evaluation of measurement instruments [6].

This study therefore aims to systematically identify HRQoL instruments used in children with DGBI, critically appraise and synthesize their measurement properties using the COSMIN methodology, and generate evidence-based recommendations for instrument selection in clinical and research settings. By consolidating fragmented measurement evidence, this work seeks to enhance standardization and comparability across studies and to strengthen the evidentiary foundation supporting clinical research, therapeutic development, regulatory evaluation, and health technology assessment in pediatric DGBI.

## Methods

### Search strategy

A systematic literature search was conducted in PubMed, Embase, and Web of Science from database inception to October 23, 2025. The search strategy combined four core concepts using Boolean operators: functional gastrointestinal disorders, now commonly referred to as disorders of gut–brain interaction (DGBI), defined according to the Rome IV criteria and including all pediatric subtypes; pediatric populations (0–18 years); health-related quality of life (HRQoL) and related terminology; and a COSMIN-recommended sensitive filter for measurement property studies [7]. The full search strategies are provided in Supplementary Material 1.

### Eligibility criteria

#### Inclusion criteria

- The instrument under evaluation was a PROM, including child self-report and/or parent proxy-report versions.
- The PROM was designed to assess HRQoL or closely related constructs in pediatric patients (0–18 years) with DGBI
- The study reported at least one measurement property of the PROM.
- The full text was available in English.

#### Exclusion criteria

- Studies included only adult populations or did not provide separable data for pediatric participants.
- Studies did not specifically report data for DGBI.
- PROMs were used solely as outcome measures without evaluation of their measurement properties.
- Duplicate publications of the same dataset were excluded; in such cases, the study providing the most comprehensive measurement property data was retained.

### Selection and data extraction

After deduplication, two reviewers independently screened titles, abstracts, and full-text articles against the predefined eligibility criteria. Discrepancies were resolved through discussion, with arbitration by a third reviewer when necessary. For all included studies, two reviewers independently extracted data using a standardized form developed in accordance with the COSMIN manual.

### Measurement property evaluation

The methodological quality of each included study was evaluated using the COSMIN Risk of Bias checklist, applied separately for each measurement property. For each property assessed, methodological quality was rated as very good, adequate, doubtful, or inadequate. The COSMIN “worst score counts” principle was applied, whereby the lowest rating of any standard within a measurement property determined the overall methodological quality.

Measurement property results were evaluated according to the COSMIN criteria for good measurement properties and classified as sufficient (+), insufficient (−), or indeterminate (?). The overall quality of evidence for each measurement property was graded using the COSMIN-recommended modified GRADE approach, considering risk of bias, inconsistency, indirectness, and imprecision. Evidence was categorized as high, moderate, low, or very low.

## Result

### Included literature

**Fig. 1:**
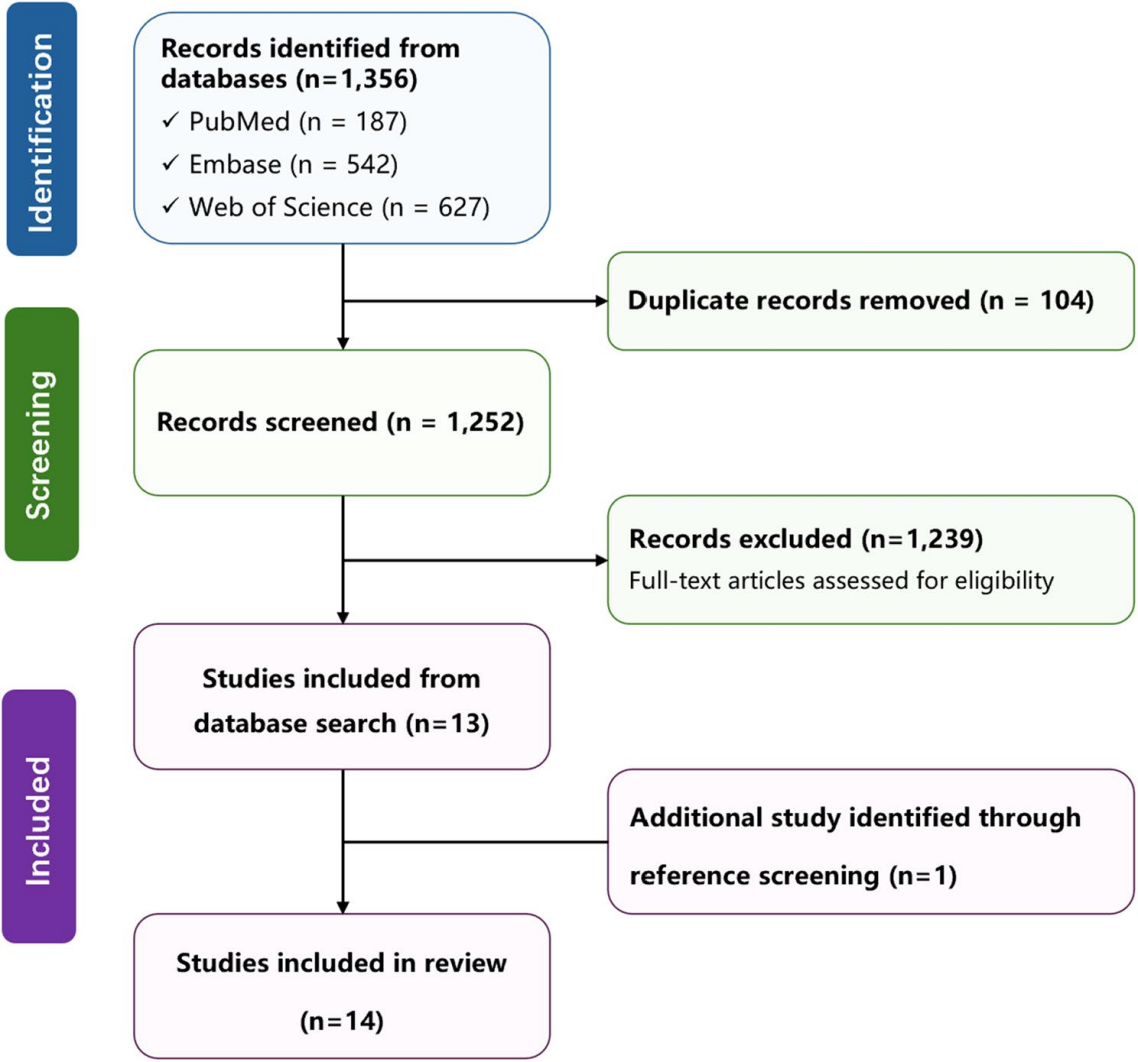
Flow diagram of study selection

The database search identified 1,356 records, including 187 from PubMed, 542 from Embase, and 627 from Web of Science. After removal of duplicates, 1,252 records remained for title and abstract screening. Following full-text assessment, 13 studies met the inclusion criteria. An additional one eligible studies were identified through manual screening of reference lists, resulting in a total of 14 studies included in the review [8]. The study selection process is shown in Fig. 1.

The included studies covered six pediatric DGBI subtypes: cyclic vomiting syndrome (CVS), infant colic (IC), functional dyspepsia (FD), irritable bowel syndrome (IBS), functional abdominal pain—not otherwise specified (FAP-NOS), and functional defecation disorders. Across these conditions, nine HRQoL instruments were identified, including generic instruments and disease-specific measures. Information on interpretability is summarized in Table 1. Risk of bias (RoB) assessments and measurement property ratings at the study level are presented in Table 2. The overall evidence synthesis for each measurement property across PROMs is shown in Table 3.

**Table 1:** Information on interpretability of PROMs

PROM	Ref #	Language	Disease	Age(N)	Distribution of scores		Missing values,%	Patients with lowest/highest scores (%)		Minimal important change (MIC)
					Mean	SD		Lowest	Highest	
**PedsQL 4.0-C**	Tarbell, 2013 [9]	English	CVS	Total (*n* = 68)	70.73	15.76	5.3	0	2.5	NR
				5–7 y (*n* = 21)	63.25	14.71	2.3	0	3.5	NR
				8–12 y (*n* = 27)	71.72	14.89	9.5	0	1.5	NR
				13–18 y (*n* = 20)	70.73	17.56	4.2	0	2.2	NR
	Varni, 2015 [3]	English	FD	5–18 y (*n* = 14)	73.7	18.2	NA	NR	NR	NR
	He, 2023 [10]	Chinese	FD	5–18 y (*n* = 1100)	64.3	8.1	NA	NR	NR	NR
	Varni, 2015 [3]	English	IBS	5–18 y (*n* = 38)	68.5	18.1	NA	NR	NR	NR
	Varni, 2015 [3]	English	FAP	5–18 y (*n* = 144)	69.3	14.9	NA	NR	NR	NR
	Hartman, 2014 [11]	Dutch	FC	Total (*n* = 198)	75.4	14.2	5.3	0	2.5	NR
				5–7 y (*n* = 85)	75.5	14.4	2.3	0	3.5	NR
				8–12 y (*n* = 67)	76.6	12.5	9.5	0	1.5	NR
				13–18 y (*n* = 46)	73.3	16.1	4.2	0	2.2	NR
	Varni, 2015 [3]	English	FC	5–18 y (*n* = 115)	71.1	18.5	NA	NR	NR	NR
**PedsQL 4.0-P**	Tarbell, 2013 [9]	English	CVS	Total (*n* = 82)	72.48	15.19	2.6	0	1.1	NR
				2–4 y (*n* = 12)	79.43	11.89	6.7	0	1.8	NR
				5–7 y (*n* = 18)	73.95	11.49	0	0	0	NR
				8–12 y (*n* = 29)	68.67	17.36	1.4	0	1.4	NR
				13–18 y (*n* = 23)	72.33	15.80	4.2	0	2.2	NR
	Varni, 2015 [3]	English	FD	2–18 y (*n* = 17)	71.4	18.1	NA	NR	NR	NR
	Varni, 2015 [3]	English	IBS	2–18 y (*n* = 42)	70.0	16.9	NA	NR	NR	NR
	Varni, 2015 [3]	English	FAP	2–18 y (*n* = 114)	68.3	17.9	NA	NR	NR	NR
	Hartman, 2014 [11]	Dutch	FC	Total (*n* = 262–266)	73.8	14.44	2.6	0	1.1	NR
				2–4 y (*n* = 56–59)	NR	NR	6.7	0	1.8	NR
				5–7 y (*n* = 87)	78.5	12.2	0	0	0	NR
				8–12 y (*n* = 63–73)	72.8	14.3	1.4	0	1.4	NR
				13–18 y (*n* = 46–47)	66.4	15.1	4.2	0	2.2	NR
	Varni, 2015 [3]	English	FC	2–18 y (*n* = 183)	71.9	20.5	NA	NR	NR	NR
**EQ-5D-Y-C**	He, 2023 [10]	Chinese	FD	5–18 y (*n* = 1100)	0.93	0.09	NA	NR	NR	NR
	van Summeren, 2018 [12]	Dutch	FC	8–17 y (*n* = 56)	84(71–92)	/	NA	NR	NR	NR
**EQ-5D-Y-P**	van Summeren, 2018 [12]	Dutch	FC	8–17 y (*n* = 56)	85(75–94)	/	NA	NR	NR	NR
**CHU-9D**	He, 2023 [10]	Chinese	FD	5–18 y (*n* = 1100)	0.92	0.08	NA	NR	NR	NR
**FIM**	Wang-Hall, 2018 [13]	English	CVS	8–18 y (*n* = 42)	69.47	16.14	NA	NR	NR	NR
**PedsQL GI Symptoms Scales-C**	Varni, 2017 [14]	English	IBS	5–18 y (*n* = 38)	68.55	18.12	NA	NR	NR	NR
	Varni, 2015 [15]	English	IBS	n = 39	61.8	16.6	NA	NR	NR	3.32
	Varni, 2017 [14]	English	FAP	5–18 y (*n* = 113)	69.24	14.88	NA	NR	NR	NR
	Varni, 2015 [15]	English	FAP	n = 115	67.5	16.8	NA	NR	NR	3.36
	Varni, 2017 [14]	English	FC	5–18 y (*n* = 108)	71.03	18.94	NA	NR	NR	NR
	Varni, 2015 [16]	English	FC	5–18 y (*n* = 116)	68.9	19.1	NA	NR	NR	3.31
**PedsQLGI Symptoms Scales-P**	Varni, 2015 [15]	English	IBS	n = 43	60.8	16.2	NA	NR	NR	3.62
	Varni, 2015 [15]	English	FAP	n = 118	66.5	16.1	NA	NR	NR	2.79
	Varni, 2015 [16]	English	FC	2–18 y (*n* = 188)	67.0	17.1	NA	NR	NR	3.42
**PedsQL GI Worry Scales-C**	Varni, 2015 [15]	English	IBS	n = 39	76.5/39.7	25.0/33.2	NA	NR	NR	Subscale1:10.61 Subscale2:12.42
	Varni, 2015 [15]	English	FAP	n = 115	82.4/46.6	23.6/32.3	NA	NR	NR	Subscale1:8.83 Subscale2:13.71
	Varni, 2015 [16]	English	FC	Total (*n* = 116)	59.5/60.2	30.3/34.7	NA	NR	NR	Subscale1:12.12 Subscale2:14.72
**PedsQL GI Worry Scales-P**	Varni, 2015 [15]	English	IBS	n = 43	74.4/44.8	22.7/30.5	NA	NR	NR	Subscale1:9.63 Subscale2:16.14
	Varni, 2015 [15]	English	FAP	n = 118	81.2/42.4	22.8/30.1	NA	NR	NR	Subscale1:7.21 Subscale2:14.12
	Varni, 2015 [16]	English	FC	Total (*n* = 188)	63.7/64.3	30.0/33.0	NA	NR	NR	Subscale1:9.51 Subscale2:12.35
**ColiQ C**	Bellaiche, 2021 [17]	French	IC	2–12w (*n* = 1298)	NR	NR	15.9	0	0	NR
**PedFCQuest-PR**	Gamarra, 2022 [18]	Portuguese	FC	5–15 y (*n* = 87)	60.9	16.2	0	NR	NR	NR
**DDL-C**	Voskuijl, 2004 [8]	Dutch	functional defecation disorders.	7–15 y (*n* = 28)	NR	NR	NA	NR	NR	NR
	Hartman, 2014 [11]	Dutch	FC	8–17 y (*n* = 56)	76(65–84)	NR	NA	NR	NR	NR
**DDL-P**	Hartman, 2014 [11]	Dutch	FC	8–17 y (*n* = 56)	78(67–85)	NR	NA	NR	NR	NR

*Abbreviations: NR: not reported; SD Standard Deviation*

**Table 2:** Results on the risk of bias (RoB) and ratings for each study on a measurement property

PROM	Ref #	Disease	Internal consistency			Reliability			Hypothesis testing: comparisons between instruments (CI) and known groups (KG)		
			N	RoB	Rating and *result*	N	RoB	Rating and *result*	N	RoB	Rating and *result*
**PedsQL 4.0-C**	Tarbell, 2013 [9]	CVS	68		? Cronbach α: 0.78–0.92	65	Very good	+ Patient–parent agreement ICC: 0.504–0.805			
	Varni, 2015 [3]	FD							KG: FD(14)/HC(936)	Doubtful	-
	He, 2023 [10]	FD							CI: 1100	Adequate	+
	Varni, 2015 [3]	IBS							KG: IBS(38)/HC(936)	Very good	+
	Varni, 2015 [3]	FAP							KG: FAP(114)/HC(936)	Very good	+
	Hartman, 2014 [11] (5–7Y)	FC	85		? Cronbach α: 0.83	85	Very good	- Patient–parent agreement ICC: 0.43(0.24–0.59)	CI: 32	Very good	+
	Hartman, 2014 [11] (8–12Y)	FC	67		? Cronbach α: 0.85	67	Very good	+ C ICC: 0.62(0.45–0.75)	CI: 64–65	Very good	+
	Hartman, 2014 [11] (13–18Y)	FC	46		? Cronbach α: 0.92	45	Very good	+ Patient–parent agreement ICC: 0.74(0.57–0.85)	CI: 44–48	Very good	+
	Varni, 2015 [3]	FC							KG: FC(115)/HC(936)	Very good	+
**PedsQL 4.0-P**	Tarbell, 2013 [9]	CVS	82		+ Cronbach α: 0.85–0.92	65	Very good	+ /Patient–parent agreement ICC: 0.504–0.805			
	Varni, 2015 [3]	FD							KG: FD(17)/HC(1106)	Doubtful	-
	Varni, 2015 [3]	IBS							KG: IBS(42)/HC(1106)	Very good	+
	Varni, 2015 [3]	FAP							KG: FAP(114)/HC(1106)	Very good	+
	Hartman, 2014 [11] (2–4Y)	FC	56–59		? Cronbach α: 0.88				CI: 56–59	Very good	+
	Hartman, 2014 [11] (5–7Y)	FC	87		? Cronbach α: 0.86	85	Very good	- Patient–parent agreement ICC: 0.43(0.24–0.59)	CI: 46	Very good	+
	Hartman, 2014 [11] (8–12Y)	FC	63–73		? Cronbach α: 0.86	67	Very good	+ Patient–parent agreement ICC:0.62(0.45–0.75)	CI: 65–71	Very good	+
	Hartman, 2014 [11] (13–18Y)	FC	46–47		? Cronbach α: 0.88	45	Very good	+ Patient–parent agreement ICC: 0.74(0.57–0.85)	CI: 45–46	Very good	+
	Varni, 2015 [3]	FC							KG: FC(183)/HC(1106)	Very good	+
**ED-5Q-Y**	He, 2023 [10]	FD							CI: 1100 KG: 213(mild)/563(Moderate)/324(Severe)	CI: Adequate KG: Very good	CI: + KG: +
	van Summeren, 2018 [12]	FC				56	Very good	+ Patient–parent agreement ICC: 0.78(0.65–0.87)			
**CHU-9D**	He, 2023 [10]	FD							CI: 1100 KG: 213(mild)/563(Moderate)/324(Severe)	CI: Adequate KG: Very good	CI: + KG: +
**FIM**	Wang-Hall, 2018 [13]	FD	42		? Cronbach α: 0.96						
**PedsQL GI Symptoms Scales -C**	Varni, 2017 [14]	IBS							CI: 38	Very good	+
	Varni, 2015 [15]	IBS	39		? Cronbach α: 0.96				KG: IBS(39)/HC(430)	Very good	+
	Varni, 2017 [14]	FAP							CI: 113	Very good	+
	Varni, 2015 [15]	FAP	39		? Cronbach α: 0.96				KG: FAP(115)/HC(430)	Very good	+
	Varni, 2017 [14]	FC							CI: 108	Very good	+
	Varni, 2015 [16]	FC	116		? Cronbach α: 0.97				KG: FC(116)/HC:(283)	Very good	+
**PedsQL GI Symptoms Scales -P**	Varni, 2015 [15]	IBS	43		? Cronbach α: 0.95				KG: IBS(43)/HC(229)	Very good	+
	Varni, 2015 [15]	FAP	43		? Cronbach α: 0.95				KG: FAP(118)/HC(229)	Very good	+
	Varni, 2015 [16]	FC	188		? Cronbach α: 0.96				KG: FC(188)/HC:(194)	Very good	+
**PedsQL GI Worry Scales -C**	Varni, 2015 [15]	IBS	39		? Subscale 1: 0.82 Subscale 2: 0.86				KG: IBS(39)/HC(430)	Very good	+
	Varni, 2015 [15]	FAP	115		? Subscale 1: 0.86 Subscale 2: 0.82				KG: FAP(115)/HC(430)	Very good	+
	Varni, 2015 [16]	FC	116		? Subscale 1: 0.84 Subscale 2: 0.82				KG: FC(116)/HC:(283)	Very good	+
**PedsQL GI Worry Scales -P**	Varni, 2015 [15]	IBS	43		? Subscale 1: 0.82 Subscale 2: 0.72				KG: IBS(43)/HC(229)	Very good	+
	Varni, 2015 [15]	FAP	118		? Subscale 1: 0.90 Subscale 2: 0.78				KG: FAP(118)/HC(229)	Very good	+
	Varni, 2015 [16]	FC	188		? Subscale 1: 0.89 Subscale 2: 0.86				KG:FC(188)/HC:(194)	Very good	+
**ColiQ C**	Bellaiche, 2021 [17]	IC	1092		? Cronbach α: 0.79	305	Adequate	+ ICC: 0.86			
**PedFCQuest-PR**	Gamarra, 2022 [18]	FC	87		? Cronbach α: 0.84–0.86						
**DDL**	Voskuijl, 2004 [8]	functional defecation disorders	27		? Cronbach α: 0.61–0.76	27	Doutful	+ ICC: 0.87			
	Hartman, 2014 [11]	FC				56	Very good	+ Patient–parent agreement ICC: 0.80(0.67–0.88)			

+ sufficient; – insufficient;  ± inconsistent; ? indeterminate

**Table 3:**
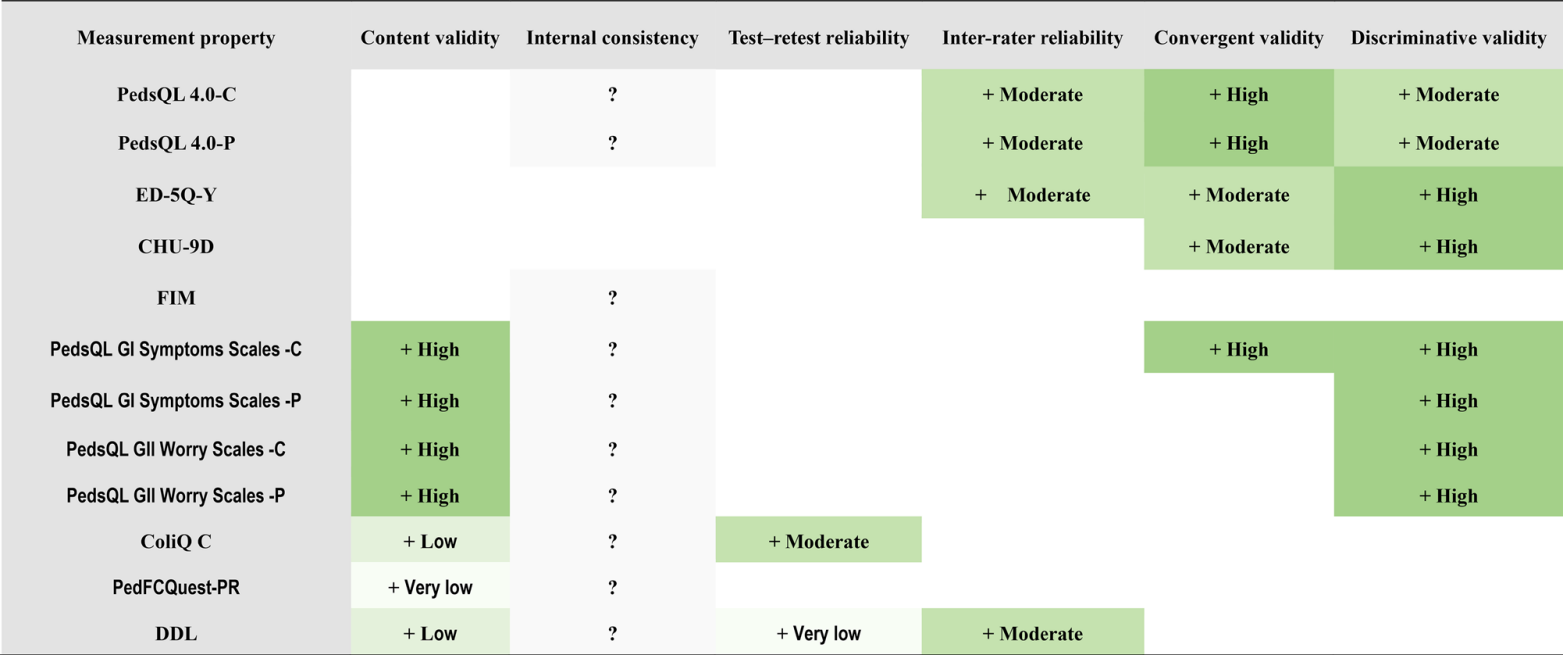
Summary of findings with the evidence for each measurement property for each PROM*

* Colors represent sufficiency of measurement properties, shading represents quality of the evidence

Green: sufficient; grey: indeterminate; darker shading: higher quality evidence

Blank space: lack of evidence

### Generic HRQoL instruments

#### PedsQL 4.0

PedsQL 4.0 was evaluated across multiple DGBI subtypes, including CVS, FD, IBS, FAP-NOS, and functional constipation (FC). It is a 23-item generic HRQoL instrument assessing physical, emotional, social, and school functioning, with age-specific child self-report and parent-proxy versions [9].

Internal consistency ranged from 0.78–0.92 for child self-reports and 0.85–0.92 for parent-proxy reports; however, absence of structural validity evidence resulted in an indeterminate rating. Inter-rater reliability was generally sufficient (most ICCs > 0.40), although in one study of children aged 2–4 years the lower bound of the ICC confidence interval fell below 0.40, leading to a moderate methodological quality rating. Convergent validity was sufficient, supported by moderate correlations with EQ-5D-Y, CHU-9D, and GI modules; methodological quality was high. Discriminative validity versus healthy controls was sufficient in both child and parent versions; one study with a small functional dyspepsia sample yielded insufficient results, leading to a moderate methodological quality rating [3].

#### EQ-5D-Y

EQ-5D-Y was evaluated in FD and FC. It measures HRQoL across five dimensions—mobility, self-care, usual activities, pain/discomfort, and anxiety/depression—each with three response levels, generating utility values based on established value sets (higher scores indicate better health) [10].

Convergent validity with PedsQL 4.0 and CHU-9D was sufficient, with moderate methodological quality. Discriminative validity across FD severity levels was sufficient, with high methodological quality. Inter-rater reliability between child and parent reports was sufficient; however, the quality of evidence was downgraded to moderate due to imprecision, as the total sample size was < 100.

#### CHU-9D

CHU-9D was evaluated in FD. It includes nine dimensions—worry, sad, pain, tired, annoyed, schoolwork/homework, sleep, daily routine, and ability to join in activities—each rated on a five-level Likert scale reflecting health status on the day of assessment [10].

Convergent validity with EQ-5D-Y and PedsQL 4.0 was sufficient, with moderate methodological quality. Discriminative validity across FD severity levels was sufficient, with high methodological quality.

#### PedsQL family impact module (PedsQL FIM)

FIM was evaluated in CVS. It consists of 36 items covering physical, emotional, social, and cognitive functioning, communication, worry, and the impacts on daily activities and family relationships. Responses are transformed into a 0–100 scale [13].

Cronbach’s α was 0.96 in children aged 8–18 years. Absence of structural validity evidence resulted in an indeterminate rating for internal consistency.

### Disease-specific HRQoL instruments

#### PedsQL Gastrointestinal Symptoms Scales

The PedsQL Gastrointestinal Symptoms Scales assess symptom-related impacts on daily functioning and HRQoL in pediatric functional and organic gastrointestinal disorders. The instrument includes 58 items across 10 symptom domains and is available in child self-report and parent-proxy versions, with age-specific response formats. Scores are transformed to a 0–100 scale, with lower scores indicating poorer symptom-specific HRQoL [15].

Content validity was sufficient for relevance, comprehensiveness, and comprehensibility, with high-quality evidence [19, 20]. Convergent validity of the child self-report version was sufficient based on correlations with the PedsQL 4.0, with high methodological quality. Cronbach’s α ranged from 0.95–0.97 (child) and 0.95–0.96 (parent); absence of structural validity evidence resulted in an indeterminate rating for internal consistency. Both child and parent versions demonstrated sufficient discriminative validity versus healthy controls, with high methodological quality. Reported MIC values ranged from 3.31–3.36 (child) and 2.79–3.62 (parent).

#### PedsQL Gastrointestinal Worry Scales

PedsQL Gastrointestinal Worry Scales include two subscales, Worry About Going Poop (5 items) and Worry About Stomach Aches (2 items), and are scored similarly to the Gastrointestinal Symptoms Scales, with scores transformed to a 0–100 scale in which lower values indicate greater gastrointestinal-specific worry [15].

Content validity was sufficient for relevance, comprehensiveness, and comprehensibility, with high quality evidence [19, 20]. Cronbach’s α ranged from 0.82–0.86 (child) and 0.72–0.90 (parent); absence of structural validity evidence resulted in an indeterminate rating for internal consistency. Both child and parent versions demonstrated sufficient discriminative validity versus healthy controls, with high methodological quality. Reported MIC values ranged from 8.83–12.12 and 12.42–14.72 for the child version (Worry About Going Poop and Worry About Stomach Aches, respectively), and from 7.21–9.63 and 12.35–16.14 for the parent version.

#### Infant Colic Questionnaire (ColiQ)

ColiQ was used in infant colic. It assesses colic-related symptoms and their impact on parents and includes 16 parent-reported items generating domain and total scores, standardized to a 0–100 scale (higher scores indicate greater symptom severity or family impact) [17].

Content validity was sufficient for relevance, comprehensiveness, and comprehensibility, with low-quality evidence. Cronbach’s α was 0.79; absence of structural validity evidence resulted in an indeterminate rating for internal consistency. Test–retest reliability over one week was sufficient (ICC = 0.86), with moderate methodological quality.

#### PedFcquest-PR

PedFCQuest-PR is a Brazilian Portuguese-language, disease-specific HRQoL instrument for functional constipation. It includes 26 items across four domains (Physical, Behavioral, Social, and School), rated on a 4-point Likert scale and transformed to a 0–100 scale, with higher scores indicating better HRQoL [18].

Content validity was rated as sufficient for relevance, comprehensiveness, and comprehensibility, with very low-quality evidence. Cronbach’s α ranged from 0.84 to 0.86; absence of structural validity evidence resulted in an indeterminate rating for internal consistency.

#### Defecation Disorder List (DDL)

DDL is a Dutch, disease-specific HRQoL instrument developed for children aged 7–15 years with constipation and/or functional non-retentive fecal soiling. The final version comprises 37 items across four domains (constipation-related, emotional functioning, social functioning, and treatment/interventions), rated on a 5-point Likert scale [8].

Content validity was rated as sufficient for relevance, comprehensiveness, and comprehensibility, with low-quality evidence. Cronbach’s α ranged from 0.61 to 0.76; absence of structural validity evidence resulted in an indeterminate rating for internal consistency. Test–retest reliability over two weeks was sufficient. However, due to risk of bias and a sample size < 50, the overall quality of evidence was downgraded to very low. Inter-rater reliability between child and parent reports was sufficient (ICC 0.67–0.88); the quality of evidence was downgraded to moderate due to imprecision, as the total sample size was < 100.

## Discussion

In this systematic review, we identified nine HRQoL instruments used in children with DGBI, including four generic instruments (PedsQL 4.0, EQ-5D-Y, CHU-9D, and PedsQL FIM) and five disease-specific instruments (PedsQL GI Symptoms Scales, PedsQL GI Worry Scales, ColiQ, PedFCQuest-PR, and DDL). Across these instruments, evidence was reported for several measurement properties, including content validity, internal consistency, test–retest reliability, inter-rater reliability, convergent validity, and discriminative validity.

Generic HRQoL instruments were not developed specifically for children with DGBI. For example, PedsQL 4.0 was originally developed in pediatric cancer populations. As children with DGBI were not part of the original target population of these instruments, content validity for DGBI populations was not assessed for generic instruments in this review. Among the generic instruments, PedsQL 4.0 showed the most comprehensive evidence across measurement properties and across multiple DGBI subtypes. This supports its use when a generic HRQoL assessment is required. The broad scope of generic instruments may limit their sensitivity to gastrointestinal-specific concerns that are central to DGBI, and improvements in gastrointestinal symptoms may not always be reflected in generic HRQoL scores.

While generic instruments provide a broad assessment of health status, disease-specific instruments aim to capture symptom-related impacts and condition-specific concerns more directly. Among these instruments, the PedsQL GI Symptoms Scales and the PedsQL GI Worry Scales showed the most robust evidence across measurement properties, including sufficient evidence for content validity. These instruments were developed in pediatric populations with gastrointestinal disorders including IBS, FC, and FAP, and their content therefore reflects the clinical experiences of common pediatric DGBI. The GI Symptoms Scales assess symptom-related functional impact, whereas the GI Worry Scales measure gastrointestinal-specific concerns and anxiety. Depending on the research objective, the two scales may be used separately or together. Both instruments were developed for children aged ≥ 2 years and are therefore not suitable for infant DGBI.

More narrowly targeted disease-specific instruments were also identified. The ColiQ, PedFCQuest-PR, and the DDL were developed for infant colic, functional constipation, and functional defecation disorders. Although these instruments provide greater disease specificity, the current evidence base remains limited. In particular, the quality of evidence supporting content validity is low, and their development within specific linguistic contexts (French, Portuguese, and Dutch) restricts broader use without appropriate translation, cultural adaptation, and validation.

Evidence on structural validity was not reported for any instrument identified in this review. Most instruments relied on classical test theory approaches, in which structural validity is typically evaluated using exploratory or confirmatory factor analysis. Such analyses require additional methodological design and statistical expertise, and are therefore not always prioritized in clinical studies where the primary objective is to apply rather than validate an instrument. Several studies reported Cronbach’s α values without examining structural validity. As COSMIN recommends interpreting internal consistency only after structural validity has been established, the results were frequently rated as indeterminate in this review.

Several additional measurement properties recommended by COSMIN were also not reported in the included studies. Cross-cultural validity and measurement invariance have not yet been examined across languages or population subgroups. Measurement error and responsiveness were also unavailable, likely reflecting the cross-sectional design of most studies and the absence of predefined MIC values. Criterion validity was not evaluated, which is common for HRQoL instruments given the lack of a true gold standard. These methodological considerations are particularly relevant in the context of DGBI, where the burden of disease is not fully captured by symptom measures alone. For example, two children may report similar abdominal pain frequency but experience markedly different functional consequences: one may maintain normal school attendance, whereas another may experience substantial disruption in learning, sleep, emotional well-being, and social participation. Likewise, reductions in symptom frequency do not necessarily translate into meaningful improvements in school attendance, psychological distress, or family burden. Given that DGBI symptoms are inherently subjective, lack objective biomarkers, and are influenced by psychosocial factors, symptom-based outcomes alone may not adequately reflect disease burden. HRQoL measures therefore provide a patient-centered assessment of the broader impact of DGBI on daily functioning and well-being.

Several limitations should be considered when interpreting the findings of this review. The number of studies evaluating HRQoL instruments in pediatric DGBI remains limited, and evidence for several measurement properties was available for only a small number of instruments, reflecting the relatively limited psychometric research conducted in this field. In addition, only English-language publications were included, which may have led to the exclusion of relevant studies reported in other languages. Furthermore, several disease-specific instruments were developed within specific linguistic contexts (e.g., French, Portuguese, and Dutch), and their broader application requires translation, cultural adaptation, and further validation in other populations.

Future research should move beyond isolated psychometric evaluations and focus on developing a more coherent framework for HRQoL measurement in pediatric DGBI. Establishing structural validity and evaluating responsiveness remain key priorities to ensure that scale scores can be interpreted meaningfully and used to detect clinically relevant change. Greater standardization of HRQoL instruments across studies may also improve comparability of outcomes in clinical research.

## Electronic supplementary material

Below is the link to the electronic supplementary material.

Supplementary Material 1

## Data Availability

No datasets were generated or analysed during the current study.

## Abbreviations

CHU-9D: Child Health Utility 9D
ColiQ: Infant Colic Questionnaire
COSMIN: COnsensus-based Standards for the selection of health Measurement INstruments
CVS: Cyclic Vomiting Syndrome
DDL: Defecation Disorder List
DGBI: Disorders of Gut–Brain Interaction
EQ-5D-Y: EuroQol 5-Dimension Youth Version
FAP-NOS: Functional Abdominal Pain, Not Otherwise Specified
FC: Functional Constipation
FD: Functional Dyspepsia
FGIDs: Functional Gastrointestinal Disorders
FIM: Family Impact Module
HRQoL: Health-Related Quality of Life
IBS: Irritable Bowel Syndrome
IC: Infant Colic
MIC: Minimal Important Change
PedFCQuest-PR: Pediatric Functional Constipation Questionnaire–Parent Report
PedsQL: Pediatric Quality of Life Inventory
PROM: Patient-Reported Outcome Measure
