# Supplementary material for: Health-related quality of life instruments in pediatric disorders of gut–brain interaction: a COSMIN-based systematic review
Source: J Patient Rep Outcomes. 2026 May 22;10:122. doi: 10.1186/s41687-026-01093-2 (PMC13381409; doi:10.1186/s41687-026-01093-2)
Supplement: Supplementary file 1 — Supplementary Material 1 [file 41687_2026_1093_MOESM1_ESM.docx]

**Search strategy**

| **PubMed** | #1 ("functional gastrointestinal disorders"[Mesh] OR "functional gastrointestinal disorders"[tiab] OR "functional GI disorders"[tiab]  OR "functional nausea and vomiting disorders"[tiab] OR "functional nausea"[tiab] OR "functional vomiting"[tiab] OR "functional abdominal pain disorders"[tiab] OR "functional abdominal pain"[tiab] OR "functional abdominal pain-not otherwise specified"[tiab]  OR "functional dyspepsia"[tiab] OR "irritable bowel syndrome"[tiab] OR IBS[tiab] OR "abdominal migraine"[tiab] OR "functional diarrhea"[tiab] OR "functional constipation"[tiab] OR "functional defecation disorders"[tiab] OR "nonretentive fecal incontinence"[tiab] OR "infant regurgitation"[tiab] OR "infant colic"[tiab] OR "infant dyschezia"[tiab] OR "cyclic vomiting syndrome"[tiab] OR "rumination syndrome"[tiab] OR "aerophagia"[tiab])  #2 ("child"[Mesh] OR child*[tiab] OR pediatric*[tiab] OR infant*[tiab])  #3 (("quality of life"[Mesh] OR "health-related quality of life"[tiab] OR HRQoL[tiab] OR "life quality"[tiab] OR "health status"[tiab])  OR ("family burden"[tiab] OR "caregiver burden"[tiab] OR "parental burden"[tiab] OR "family impact"[tiab] OR "family functioning"[tiab] OR "parent stress"[tiab] OR "caregiver stress"[tiab] OR "caregiver quality of life"[tiab]))  #4 (instrumentation[sh] OR methods[sh] OR “Validation Study“[pt] OR “Comparative Study”[pt] OR “psychometrics”[MeSH]  OR psychometr*[tiab] OR clinimetr*[tw] OR clinometr*[tw] OR “outcome assessment, health care“[MeSH]  OR “outcome assessment”[tiab] OR “outcome measure*”[tw] OR “observer variation”[MeSH]  OR “observer variation”[tiab] OR “Health Status Indicators”[Mesh]  OR “reproducibility of results”[MeSH] OR reproducib*[tiab] OR “discriminant analysis”[MeSH]  OR reliab*[tiab] OR unreliab*[tiab] OR valid*[tiab] OR “coefficient of variation”[tiab]  OR coefficient[tiab] OR homogeneity[tiab] OR homogeneous[tiab]  OR “internal consistency”[tiab] OR (cronbach*[tiab] AND (alpha[tiab] OR alphas[tiab]))  OR (item[tiab] AND (correlation*[tiab] OR selection*[tiab] OR reduction*[tiab]))  OR agreement[tw] OR precision[tw] OR imprecision[tw] OR “precise values”[tw]  OR test-retest[tiab] OR (test[tiab] AND retest[tiab])  OR (reliab*[tiab] AND (test[tiab] OR retest[tiab])) OR stability[tiab]  OR interrater[tiab] OR inter-rater[tiab] OR intrarater[tiab] OR intra-rater[tiab]  OR interobserver[tiab] OR inter-observer[tiab] OR intraobserver[tiab] OR intra-observer[tiab]  OR interassay[tiab] OR inter-assay[tiab] OR intraassay[tiab] OR intra-assay[tiab]  OR kappa[tiab] OR kappas[tiab] OR repeatab*[tw]  OR ((replicab*[tw] OR repeated[tw]) AND (measure[tw] OR measures[tw] OR findings[tw] OR result[tw] OR results[tw] OR test[tw] OR tests[tw]))  OR generaliza*[tiab] OR concordance[tiab] OR (intraclass[tiab] AND correlation*[tiab])  OR discriminative[tiab] OR “known group”[tiab] OR “factor analysis”[tiab]  OR “factor analyses”[tiab] OR “factor structure”[tiab] OR “factor structures”[tiab]  OR dimension*[tiab] OR subscale*[tiab]  OR (multitrait[tiab] AND scaling[tiab] AND (analysis[tiab] OR analyses[tiab]))  OR “item discriminant”[tiab] OR “interscale correlation*”[tiab]  OR “standard error of measurement”[tiab] OR sensitiv*[tiab] OR responsive*[tiab]  OR interpretab*[tiab] OR ((minimal[tiab] OR clinically[tiab]) AND (important[tiab] OR significant[tiab]) AND (change[tiab] OR difference[tiab]))  OR “ceiling effect”[tiab] OR “floor effect”[tiab] OR Rasch[tiab]  OR “Differential item functioning”[tiab] OR DIF[tiab] OR “item response model”[tiab] OR IRT[tiab]  OR “computer adaptive testing”[tiab] OR “item bank”[tiab] OR “cross-cultural equivalence”[tiab])  #5 (“addresses”[Publication Type] OR “biography”[Publication Type] OR “case reports”[Publication Type] OR “comment”[Publication Type] OR “directory”[Publication Type] OR “editorial”[Publication Type] OR “festschrift”[Publication Type] OR “interview”[Publication Type] OR “lectures”[Publication Type] OR “legal cases”[Publication Type] OR “legislation”[Publication Type] OR “letter”[Publication Type] OR “news”[Publication Type] OR “newspaper article”[Publication Type] OR “patient education handout”[Publication Type] OR “popular works”[Publication Type] OR “congresses”[Publication Type] OR “consensus development conference”[Publication Type] OR “consensus development conference, nih”[Publication Type] OR “practice guideline”[Publication Type]) NOT (“animals”[MeSH Terms] NOT “humans”[MeSH Terms])  #6=(#1 AND #2 AND #3 AND #4) NOT #5  **Total:187** |
| --- | --- |
| **Web of Science** | #1 TS=("functional gastrointestinal disorders" OR "functional GI disorders"  OR "functional nausea and vomiting disorders" OR "functional nausea" OR "functional vomiting"  OR "functional abdominal pain disorders" OR "functional abdominal pain" OR "functional abdominal pain-not otherwise specified"  OR "functional dyspepsia" OR "irritable bowel syndrome" OR IBS  OR "abdominal migraine" OR "functional diarrhea" OR "functional constipation"  OR "functional defecation disorders" OR "nonretentive fecal incontinence"  OR "infant regurgitation" OR "infant colic" OR "infant dyschezia"  OR "cyclic vomiting syndrome" OR "rumination syndrome" OR "aerophagia")  #2 TS=(child* OR pediatric* OR infant*)  #3 TS=("quality of life" OR "health-related quality of life" OR HRQoL OR "life quality" OR "health status"  OR "family burden" OR "caregiver burden" OR "parental burden" OR "family impact" OR "family functioning"  OR "parent stress" OR "caregiver stress" OR "caregiver quality of life")  #4 TS=(  instrumentation OR methods OR "validation study" OR "comparative study" OR psychometrics OR psychometr*  OR clinimetr* OR clinometr* OR "outcome assessment" OR "outcome measure*" OR "observer variation"  OR "Health Status Indicators" OR reproducib* OR reliab* OR valid* OR "coefficient of variation" OR "internal consistency"  OR (cronbach* AND (alpha OR alphas)) OR (item AND (correlation* OR selection* OR reduction*))  OR agreement OR precision OR imprecision OR "precise values" OR test-retest OR stability  OR interrater OR inter-rater OR intrarater OR intra-rater OR intertester OR inter-tester OR intratester OR intra-tester  OR interobserver OR inter-observer OR intraobserver OR intra-observer OR intertechnician OR inter-technician  OR intratechnician OR intra-technician OR interexaminer OR inter-examiner OR intraexaminer OR intra-examiner  OR interassay OR inter-assay OR intraassay OR intra-assay OR interindividual OR inter-individual OR intraindividual OR intra-individual  OR interparticipant OR inter-participant OR intraparticipant OR intra-participant OR kappa OR kappas  OR repeatab* OR ((replicab* OR repeated) AND (measure OR measures OR findings OR result OR results OR test OR tests))  OR generaliza* OR generalisa* OR concordance OR (intraclass AND correlation*) OR discriminative OR "known group"  OR "factor analysis" OR "factor analyses" OR "factor structure" OR "factor structures"  OR dimension* OR subscale* OR (multitrait AND scaling AND (analysis OR analyses)) OR "item discriminant"  OR "interscale correlation*" OR error OR errors OR "individual variability" OR "interval variability" OR "rate variability"  OR (variability AND (analysis OR values)) OR (uncertainty AND (measurement OR measuring))  OR "standard error of measurement" OR sensitiv* OR responsive* OR (limit AND detection) OR "minimal detectable concentration"  OR interpretab* OR ((minimal OR minimally OR clinical OR clinically) AND (important OR significant OR detectable) AND (change OR difference))  OR (small* AND (real OR detectable) AND (change OR difference)) OR "meaningful change" OR "minimal important change"  OR "minimal important difference" OR "minimally important change" OR "minimally important difference"  OR "minimal detectable change" OR "minimal detectable difference" OR "minimally detectable change" OR "minimally detectable difference"  OR "minimal real change" OR "minimal real difference" OR "minimally real change" OR "minimally real difference"  OR "ceiling effect" OR "floor effect" OR "item response model" OR IRT OR rasch OR "differential item functioning" OR DIF  OR "computer adaptive testing" OR "item bank" OR "cross-cultural equivalence"  )  #5=#1 AND #2 AND #3 AND #4  **Total:627** |
| **Embase** | #1 ('functional gastrointestinal disorder':de OR 'functional gastrointestinal disorder':ti,ab,kw OR FGID:ti,ab,kw  OR 'functional dyspepsia':de OR 'functional dyspepsia':ti,ab,kw OR FD:ti,ab,kw  OR 'irritable bowel syndrome':de OR 'irritable bowel syndrome':ti,ab,kw OR IBS:ti,ab,kw  OR 'functional abdominal pain':ti,ab,kw OR FAP:ti,ab,kw  OR 'abdominal pain related functional gastrointestinal disorder':ti,ab,kw OR 'AP-FGID':ti,ab,kw)  #2 (child*:ti,ab,kw OR pediatric*:ti,ab,kw OR paediatric*:ti,ab,kw OR adolescent*:ti,ab,kw  OR infant*:ti,ab,kw OR toddler*:ti,ab,kw)  #3 ('quality of life':de OR 'health related quality of life':ti,ab,kw OR HRQoL:ti,ab,kw  OR 'life quality':ti,ab,kw OR 'patient reported outcome':ti,ab,kw OR PRO:ti,ab,kw  OR 'family burden':ti,ab,kw OR 'caregiver burden':ti,ab,kw OR 'family impact':ti,ab,kw  OR 'parental stress':ti,ab,kw OR 'family functioning':ti,ab,kw)  #4 (instrumentation:ti,ab,kw OR methods:ti,ab,kw  OR 'validation study':ti,ab,kw OR 'comparative study':ti,ab,kw  OR psychometr*:ti,ab,kw OR clinimetr*:ti,ab,kw OR clinometr*:ti,ab,kw  OR 'outcome assessment':ti,ab,kw OR 'outcome measure*':ti,ab,kw  OR 'observer variation':ti,ab,kw OR reproducib*:ti,ab,kw  OR reliab*:ti,ab,kw OR valid*:ti,ab,kw OR 'internal consistency':ti,ab,kw  OR (cronbach* NEAR/3 (alpha OR alphas)):ti,ab,kw  OR (test NEAR/3 retest):ti,ab,kw  OR stability:ti,ab,kw OR agreement:ti,ab,kw OR precision:ti,ab,kw  OR imprecision:ti,ab,kw OR repeatab*:ti,ab,kw OR concordance:ti,ab,kw  OR (intraclass NEAR/3 correlation*):ti,ab,kw  OR discriminative:ti,ab,kw OR dimension*:ti,ab,kw OR subscale*:ti,ab,kw  OR sensitiv*:ti,ab,kw OR responsive*:ti,ab,kw  OR 'ceiling effect':ti,ab,kw OR 'floor effect':ti,ab,kw  OR 'factor analysis':ti,ab,kw OR rasch:ti,ab,kw OR IRT:ti,ab,kw  OR 'differential item functioning':ti,ab,kw OR DIF:ti,ab,kw  OR 'computer adaptive testing':ti,ab,kw OR 'item bank':ti,ab,kw  OR 'cross cultural equivalence':ti,ab,kw)  #5 (address:ti,ab,kw OR biography:ti,ab,kw OR 'case report':ti,ab,kw OR comment:ti,ab,kw  OR editorial:ti,ab,kw OR letter:ti,ab,kw OR news:ti,ab,kw  OR 'practice guideline':ti,ab,kw)  #6=(#1 AND #2 AND #3 AND #4) NOT #5  **Total:542** |

*Article title.* Health-related quality of life instruments in pediatric disorders of gut–brain interaction: a COSMIN-based systematic review

*Journal name.* Journal of Patient-Reported Outcomes
